# Diagnostic Ureteroscopy in CT Urography-Diagnosed Upper Tract Urothelial Carcinoma: Delay in Definitive Treatment and Increased Intravesical Recurrence

**DOI:** 10.7759/cureus.15775

**Published:** 2021-06-20

**Authors:** Hadi SHSM, Elizabeth Bright, Mark Mantle, Nicholas Munro, Omar Fahmy

**Affiliations:** 1 Urology, Cornwall Royal Hospital, Truro, GBR; 2 Urology, Universiti Putra Malaysia, Serdang, MYS

**Keywords:** upper tract urothelial carcinoma, ureteroscopy, intravesical recurrence, nephroureterectomy, bladder cancer

## Abstract

Purpose

To investigate the effect of diagnostic ureteroscopy (URS) on the delay to surgical treatment of upper tract urothelial carcinoma (UTUC) detected by imaging and the risk of intravesical recurrence.

Materials and methods

We undertook a retrospective case-note analysis of all patients who underwent radical nephroureterectomy (NUU) from November 2012 to July 2019. We identified those who underwent diagnostic ureteroscopy prior to NUU as Group 1 and those who did not undergo diagnostic URS as Group 2. Perioperative and pathological parameters were compared between both groups. Kaplan-Meier and Log-Rank analyses were used to compare delay to NUU and the intravesical recurrence (IVR) free survival. Cox regression models were employed to analyze the risk factors of intravesical recurrence.

Results

Out of 69 patients with a mean age of 71.3 years and a mean follow-up of 48.5 months, 49 (71%) underwent URS while 20 (29%) did not. The mean time between the computerized tomography urography (CTU) and surgery was 86 days with URS and 59 days in the control groups(p=0.007). Intravesical recurrence in year one postoperatively was 28.2 % in the URS group vs 5.9% in the control group (p=0.04). The Kaplan-Meier curve showed improved, yet insignificant, IVR-free survival for the control group (Log-Rank p-value=0.21). In multivariate Cox regression analysis, concomitant bladder carcinoma was an independent risk factor for IVR (HR, 15.01; 95%CI, 3.311 - 68.07; p=0.0004). Intravesical mitomycin-c was a protective factor (HR 0.154; 95%CI 0.025 - 0.922; p=0.040).

Conclusion

In our retrospective single-unit study, diagnostic ureteroscopy for CTU-detected upper tract urothelial carcinoma delayed definitive surgical treatment. Furthermore, it was associated with a significantly increased risk of early intravesical recurrence. URS can provide useful information and reassurance prior to major surgery but must be used with caution in light of these findings.

## Introduction

Upper tract urothelial carcinoma (UTUC) is a rare malignancy, constituting only 5%-10% of all urothelial carcinomas [1]. However, more than half of patients have an invasive disease at presentation [2]. Radical nephroureterectomy (RNU) is the gold standard treatment for patients with a high risk of metastatic disease, who represent the majority of the patients [3]. Techniques to determine disease stage and depth of invasion at presentation are limited. Computerized tomography urography (CTU) is currently the most accurate imaging modality to diagnose UTUC, with a sensitivity and specificity of 92% and 95%, respectively [4]. Diagnostic ureteroscopy (URS) can be performed to resolve inconclusive CTU findings [3]; however, many urologists perform URS as a confirmatory tool for UTUC that is clearly diagnosed on CTU [5].

Diagnostic URS prior to NUU has both benefits and risks to the patient. One benefit of URS is the possibility to perform a biopsy and obtain histopathological confirmation of UTUC, as well as visually identify a presumed tumor. It is relatively unusual to submit patients to major cancer surgery without visual or histological confirmation of the diagnosis, i.e. to rely on the CTU alone. However, URS has the potential to increase time delay to RNU and delay definitive treatment and thus adversely affect the overall outcome [6]. A delay in definitive surgery from diagnosis is thought to be a risk factor for adverse outcomes; however, the threshold of significant delay is controversial and has been proposed to range from 30 to 90 days [3]. Additionally, It has been proposed that manipulation of the tumor during URS might increase the risk of intravesical recurrence (IVR) of urothelial cancer in the future [5,7].

In our unit, we submit a proportion of those with putative UTUC to URS after individual case reviews at our multidisciplinary meeting (MDT). We wanted to assess the risks and benefits of doing so and better advise our patients for the future. Specifically, this study was undertaken to investigate the potential delay of diagnostic URS on the definitive surgical treatment for patients with UTUC detected by CTU. In addition, we assessed whether performing diagnostic URS in this situation increased IVR.

## Materials and methods

Study population

After obtaining ethical committee approval, we undertook a retrospective analysis of all patients receiving RNU for UTUC at one institution between November 2012 and July 2019. Patients with normal findings on CTU were excluded from the analysis.

Management

All patients had either open or laparoscopic RNU with excision of the bladder cuff. Diagnostically rigid or flexible URS with or without biopsy was performed in selected patients after discussion at a multidisciplinary cancer meeting (MDT). Diagnostic cystoscopy was performed routinely for all patients as part of the diagnostic protocol. Postoperative intravesical mitomycin-c or systemic adjuvant chemotherapy were given on a case-by-case basis after ratification at the MDT.

Follow-up

All patients underwent radiological surveillance and regular cystoscopic examination post-RNU according to the European Association of Urology (EAU) Guidelines [4]. Both the development of metastatic disease and death due to UTUC were recorded with reference to duration since RNU.

Data analysis

We retrospectively reviewed clinical data and recorded the patient’s characteristics, pathological features of the UTUC, the time interval between the CTU and the date of RNU. Follow-up and survival data were recovered.

Patients who underwent diagnostic URS were assigned as the study group. Patients who did not undergo URS were assigned as the control group. The primary outcomes of this analysis were: a) Impact of diagnostic URS on delay to surgery and b) Impact on intravesical recurrence incidence.

Statistical analysis

For this study, statistical analysis was performed using the JMP V.15.2.1 software (SAS Inc., Cary, NC). All data were presented as median and range for continuous variables and as absolute numbers with percentages for categorical variables. The Mann-Whitney U and Pearson x2 tests were employed to compare the continuous and categorical variables, respectively. Kaplan Meier curves were used to present the survival data with the log-rank test for comparison. Univariate and multivariate Cox regression analyses were conducted to assess the risk factors of intravesical recurrence. A p-value of <0.05 was regarded as significant.

## Results

We identified 70 patients who underwent RNU. One patient was excluded from the study with normal findings on CTU and of the remaining 69 patients, 49 (71%) underwent diagnostic URS. The remaining 20 patients (29%) were assigned as a control group. The patient’s perioperative characteristics and the comparison between the two groups are presented in Table 1. There was no significant difference between both groups, except in gender (71% male and 29% female vs 45% and 55%, respectively, p=0.04) and in adjuvant chemotherapy administration (8.3% in the URS group vs 31.6% in the control group, p=0.02).

**Table 1:** Perioperative characteristics of included patients *significant p-value

Variable	All patients(n=69)	URS group(n=49)	Non-URS group (n=20)	P-value
Age	71.3 ± 8.4	71 ± 8	72 ± 10	0.94
Male	25	35 (71%)	9 (45%)	0.04*
Female	25	14 (29%)	11(55%)	
Follow-up	48.5 ± 25	52 ± 24	36 ± 23	0.10
Laparoscopic	63	46 (94%)	17 (85%)	0.25
Open	6	3 (6.0%)	3 (15%)	
Kidney	39	26 (53%)	13 (65%)	0.48
Proximal ureter	3	2 (4.0%)	1 (5.0%)	
Mid ureter	6	4 (8.0%)	2 (10%)	
Distal ureter	17	13 (27%)	4 (20%)	
Multiple	4	4 (8.0%)	0 (0.0%)	
Hydronephrosis	28	19 (39%)	9 (45%)	0.67
No hydronephrosis	41	30 (61%)	11 (55%)	
Concomitant bladder Ca	7	5 (10%)	2 (10%)	0.97
No bladder Ca	62	44 (90%)	18 (90%)	
Urothelial Ca	67	48 (98%)	19 (95%)	0.53
Benign lesion	2	1 (2.0%)	1 (5.0%)	
Pathological stage ≥ T3	31	21 (44%)	10 (53%)	0.51
≤ T2	36	27 (56%)	9 (47%)	
Grade 1	2	2 (4.0%)	0 (0.0%)	0.24
Grade 2	26	21 (44%)	5 (26%)	
Grade 3	39	25 (52%)	14 (74%)	
Lymph node involved	2	1 (2.0%)	1 (5.0%)	0.53
No nodal involvement	65	47 (98%)	18 (95%)	
Positive surgical margin	12	8 (17%)	4 (21%)	0.67
Negative surgical margin	55	40 (83%)	15 (79%)	
Mitomycin-C given	18	11 (23%)	7 (37%)	0.27
No mitomycin C	49	37 (77%)	12 (63%)	
Adjuvant chemo given	10	4 (8.3%)	6 (31.6%)	0.02*
No adjuvant chemo	57	44 (91.7%)	13 (68.4%)	

There was no change in the treatment strategy for any patient who underwent URS. One patient in each group had benign disease based on the histopathology report of the post-RNU specimen.

Impact of URS on delay to definitive surgery

**Figure 1:**
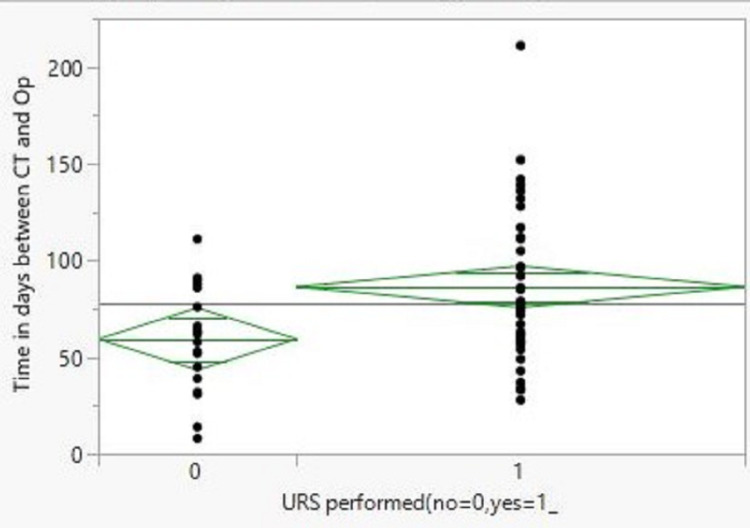
Comparison of the duration between the CTU and RNU in days CTU: computerized tomography urography; RNU: radical nephroureterectomy

There was a significant delay in RNU due to URS compared to the control group but none beyond the recommended 90-day target (Figure 1). The mean time ± SD between the CTU and RNU was 86 ± 37 days compared to 59 ± 27 in the control group (p=0.007). Seven patients were excluded from the delay analysis, as they had their surgery delayed for reasons unrelated to the URS findings.

Intravesical recurrence

**Figure 2:**
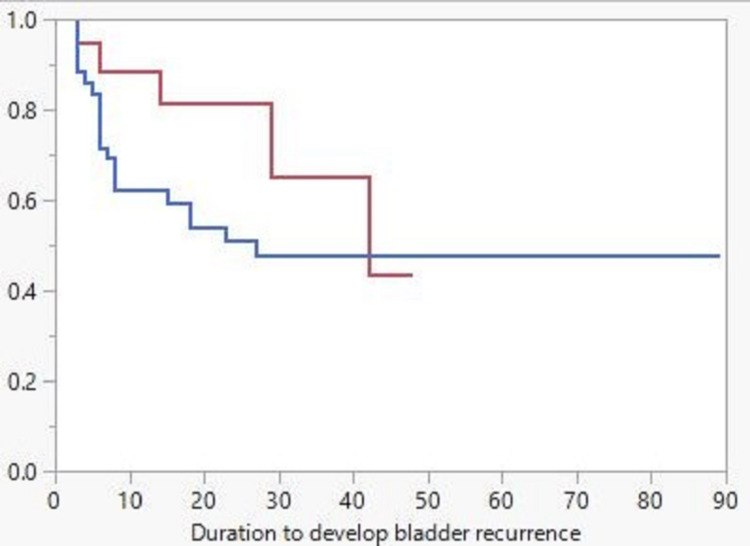
Kaplan-Meier curve for IVR-free survival; red line for the non-URS group and blue line for the URS group IVR: intravesical recurrence; URS: ureteroscopy

Fifty-nine (59) patients with a mean follow-up of 48.5 months were included in intravesical recurrence-free survival analysis (7 patients with concomitant bladder cancer, 2 patients with benign disease, and 1 patient who died from postoperative complication were excluded). The Kaplan-Meier curve (Figure 2) displayed better intravesical recurrence-free survival for the control group as compared to the URS group; however, the difference is insignificant (log-rank p-value=0.21). The incidence of recurrence within the first 12 months postop was 28.2% in the URS group compared to 5.9% in the control group (p=0.04). Overall recurrence in the URS group was 42.1% compared to 23.5% in the control group (p=0.18).

All the available variables, including the presence of concomitant bladder urothelial carcinoma, were included in univariate and multivariate Cox regression analyses for intravesical recurrence (Table 2). In univariate analysis, concomitant bladder UC was significantly associated with a higher risk of recurrence (HR, 4.8; 95%CI, 1.868-12.34; p=0.001). In multivariate analysis, concomitant bladder UC was an independent risk factor for intravesical recurrence (HR, 15.01; 95%CI, 3.311 - 68.07; p=0.0004). In addition, single-dose intravesical mitomycin-c was significantly associated with lower risk in multivariate analysis (HR 0.154; 95%CI 0.025 - 0.922; p=0.040).

**Table 2:** Cox regression model for risk factors of intravesical recurrence *significant p-value

Variable	Univariate			Multivariate		
	HR	95%CI	P value	HR	95%CI	P value
Age	0.984	0.943 - 1.029	0.471	0.961	0.892 - 1.035	0.299
Sex (Male vs Female)	1.503	0.631 - 3.582	0.357	2.694	0.763 - 9.514	0.123
Stage (≥T3 vs	1.314	0.597 - 2.889	0.497	0.456	0.093 - 2.219	0.330
Grade (3 vs 1or2)	1.549	0.699 - 3.429	0.280	3.001	0.869 - 10.35	0.082
Hydronephrosis	0.802	0.357 - 1.802	0.594	0.725	0.239 - 2.201	0.570
Focality (Multi vs Uni)	2.310	0.539 - 9.902	0.259	3.217	0.551 - 18.76	0.194
Delay of NU	1.819	0.759 - 4.358	0.179	0.777	0.234 - 2.571	0.679
Concomitant Bl UC	4.800	1.868-12.34	0.001*	15.01	3.311 - 68.07	0.0004*
Diag. URS (yes vs no)	1.809	0.681 - 4.802	0.234	1.840	0.482 - 7.019	0.372
Surgical margin	1.916	0.715 - 5.129	0.195	1.775	0.299 - 10.52	0.527
Procedure (Lap vs open)	2.358	0.319 - 17.41	0.400	2.265	0.163 - 31.34	0.541
Mitomycin C	0.752	0.299 - 1.890	0.544	0.154	0.025 - 0.922	0.040*
Adjuvant Chemotherapy	0.932	0.275 - 3.154	0.910	1.256	0.174 - 9.044	0.820

## Discussion

Upper tract urothelial carcinoma is a rare, but challenging, form of urological cancer [8-9]. Assessment of the T stage by CTU is difficult and may underestimate the actual depth of underlying muscle involvement [10]. Studies have suggested more than 95% diagnostic accuracy for tumors that are obviously locally advanced, at least >pT3 tumors [11].

Diagnostic ureteroscopy may be performed to allow complete inspection of the collecting system and obtain a tissue biopsy. This may be performed for cases where the preoperative CT scan is unable to convincingly diagnose the presence of a urothelial carcinoma as the cause of a filling defect or collecting system mass. Furthermore, URS also lacks the ability of accurate staging and depths of invasion assessment. Diagnostic biopsies obtained are often small volume tissue samples that do not accurately represent the tumor [12].

This study displayed that, despite having a prior diagnostic ureteroscopy, all patients eventually underwent radical nephroureterectomy. There was a significant delay in performing RNU as compared to the patients who did not have URS; however, this was not beyond 90 days. Yet, comparing the two groups, there was no significant difference in the final tumor stage on histology.

One of the challenges in UTUC treatment is the risk of IVR that can affect up to 47% of patients [6]. A common concern with a prior diagnostic ureteroscopy is the incidence of increased IVR [6]. However, a few studies refute that risk [13]. This is postulated to occur due to tumor manipulation and the increased intrarenal pressures during ureteroscopy, which can lead to the shedding of cancer cells and implantation into the bladder [14]. This theory might explain our findings that the IVR was more obvious and significantly higher in the URS group during the first year post-RNU.

Our analysis for IVR included other characteristics such as gender, tumor location, upper tract tumor grade, unifocality or multifocality, and if there was any delay to definitive extirpative surgery. Surgical technique, i.e. open vs laparoscopic approach, also did not affect the rates of intravesical recurrence. Though not statistically significant, this finding is in line with multiple studies performed to investigate this, including the systemic analysis by Marchioni et al. in 2017, which found a significant incidence of intravesical recurrence following diagnostic ureteroscopy [6].

We found in our cohort of patients that the presence of concomitant bladder urothelial carcinoma was the single most predictive factor for intravesical recurrence, which is not unexpected. However, this was independent of the fact if the patient had a prior diagnostic ureteroscopy. Some authors have found a definite correlation between the presence of concomitant bladder urothelial carcinoma as a predictive factor for intravesical recurrence [15], whereas others did not find a significant correlation [16-17].

In our study, a single instillation of intravesical chemotherapy significantly reduced the incidence of intravesical recurrence. This underlies the principles of reducing circulating tumor cells and thus the implantation of tumor cells into the bladder, which was proven by a randomized controlled trial [18].

This is a retrospective review with all the limitations this implies. Notably, the selection of patients who will undergo URS and those who will not was undertaken on an individual basis prospectively by an individual urologist. This review may be subject to the risk of subtle bias with treatment selection for our comparison patient groups.

A further limitation of our study is the heterogeneity of URS techniques. There is variation in the use of semi-rigid or flexible scopes, the length of the procedure, and the use of access sheath. There is also variation in the use of perioperative ureteric stenting and the inherent impact of a stent towards disseminating tumor cells from a more proximal location to the bladder.

This study was designed to clarify the role of diagnostic URS. The current practice of reserving diagnostic URS only for cases that pose a diagnostic dilemma seems reasonable. In this study, given only two non-cancer diagnoses from the overall cohort, which constitutes approximately 3% of patients, and with the anticipation that future research and investigative techniques will continue to improve, it may be reasonable to rely on CTU only for the majority of cases.

Only obtaining a definitive tissue diagnosis prior to RNU might not be a strong enough indication for URS when the patient can be assigned as high risk based on other factors such as multifocality and size of the lesion from the CTU. A fine balance needs to be struck between the possibility of benign lesion or misdiagnosis versus the increased risk of intravesical recurrence and potential delay to definitive extirpative surgery.

## Conclusions

In our retrospective single-unit study, diagnostic ureteroscopy for CTU-detected upper tract urothelial carcinoma delayed definitive surgical treatment. Furthermore, it was associated with an increased risk of early intravesical recurrence in the URS group as compared to the control group. While URS can provide useful information and reassurance prior to major surgery, it must be used with caution in light of these findings.
